# Postpartum Mood Disorders and Thyroid Autoimmunity

**DOI:** 10.3389/fendo.2017.00091

**Published:** 2017-05-04

**Authors:** Maria Le Donne, Carmela Mento, Salvatore Settineri, Alessandro Antonelli, Salvatore Benvenga

**Affiliations:** ^1^Unit of Gynecology and Obstetrics, Department of Human Pathology in Adulthood and Childhood “G. Barresi”, University of Messina, Messina, Italy; ^2^Department of Cognitive Sciences, Psychology, Educational and Cultural Studies (COSPECS), University of Messina, Messina, Italy; ^3^Department of Biomedical and Dental Sciences and Morphofunctional Imaging (BIOMORF), University of Messina, Messina, Italy; ^4^Department of Clinical and Experimental Medicine, University of Pisa, Pisa, Italy; ^5^Department of Clinical and Experimental Medicine, University of Messina, Messina, Italy; ^6^Interdept. Program of Molecular & Clinical Endocrinology and Women’s Endocrine Health, University Hospital Policlinico G. Martino, Messina, Italy

**Keywords:** postpartum, mood disorders, autoimmunity, thyroid hormones, depression

## Introduction

Because of the rapid emotional and endocrine changes in the postpartum period (1), postpartum mood disorders represent the most frequent form of maternal psychiatric morbidity (2–4). Postpartum mood disorders vary from a mild form of transient depression (maternity blues) to full-blown postpartum depression and severe psychosis (5, 6). Postpartum depression affects 10–30% of women within 1 year after delivery (7), and its risk is measurable already at 3 (8) or 7 days (2) postpartum. This risk predicts depression development in the following months (9, 10).

Thyroid function abnormalities exhibit comorbidity with various psychiatric disorders, including maternal depression. There are one-tenth of a million studies on mood disorders, but fewer than 5,000 (3.9% of almost 125,000) concern mood disorders in the postpartum period. Similarly, studies on autoimmune thyroid disease are almost 20,000, but only 72 (3.7% of 19,360) concern postpartum mood disorders and thyroid disorders, and merely 5 focus on postpartum mood disorders and thyroid autoimmunity. Thus, we hope that our opinion will stimulate interest.

## Postpartum Affective Disorders and Thyroid Dysfunction

Affective disorders and autoimmune thyroiditis are well known to affect women during puerperium. Up to 23% of all new mothers experiences thyroid dysfunction postpartum (11), compared with a prevalence of 3–4% in the general population (12). Maternal thyroid autoimmunity refers to the detection of thyroid autoantibodies against thyroperoxidase (TPOAb) and/or thyroglobulin (TgAb) in combination with normal thyroid function, and it has been reported to affect between 8 and 14% women in reproductive age (13). Concerning positivity for TPOAb, approximately 10% of pregnant women are TPOAb positive, and around one-third to half of them will develop postpartum thyroiditis (PPT) within 12 months after delivery (11, 14).

At least 2–3% of women have some form of thyroid dysfunction during pregnancy, and 10–17% of women have thyroid autoimmune disease despite euthyroidism (15, 16). Thyroid dysfunction in pregnancy is classified as (i) primary overt hypothyroidism (elevated TSH and a decreased FT4 during gestation with both concentrations outside the trimester-specific reference ranges) or subclinical hypothyroidism (with a TSH elevated beyond the upper limit of the pregnancy-specific reference range); (ii) hyperthyroidism (autoimmune Graves’ disease or gestational transient thyrotoxicosis) (17); and (iii) positivity of thyroid autoantibodies, which increases the risk of thyroid dysfunction following delivery and during the postpartum period. PPT is an inflammatory autoimmune condition, which occurs within the first year after delivery or miscarriage (a period when the immunosuppressive effect of pregnancy disappears), in women who were euthyroid prior to pregnancy (16, 18).

## Mood Disorders and Thyroid Autoimmunity in General Population

The association between subclinical hypothyroidism, with and without raised TPOAb, and well-being or depression is still controversial, in spite of many studies on this topic. Before moving to the abnormal setting, it is appropriate to mention the prevalence of thyroid autoantibodies in the general population and their association with mood disorders outside of the postpartum context.

An interaction between thyroid autoantibodies and mood disorders was first valuated in the early 1980s (19). Prevalence rates of thyroid autoantibodies in the general population vary widely, depending on several reasons, including sex and age distribution, geographic origin, variations in the cutoff level for antibody positivity (20–22). In an Italian survey, the overall prevalence of thyroid autoantibodies, using a cutoff of 1:100 for both microsomal antibodies and/or thyroid autoantibodies was 12.6% (females, 17.3%; males, 7.0%) (23). The reported prevalence in the United States healthy population was 13% for TPOAb (threshold at ≥0.5 IU/ml) and 11.5% for TgAb (threshold at ≥1.0 IU/ml) (21). Overall, the association of circulating thyroid autoantibodies with not otherwise specified mood disorders cannot be considered clearly established.

On the contrary, increased prevalence of circulating thyroid autoantibodies is shown in the following forms of mood disorders: treatment-refractory cases (24, 25), severe (26–28), atypical depression (29), anxiety disorders or mood disorders (30), and depression during early gestation, postpartum, and perimenopause (31–36).

In a population-based study, depression and anxiety were not associated with thyroid autoantibodies (37). In 2,049 subjects, autoimmune markers (TPOAb, anti-nuclear autoantibodies, and several other autoantibodies) were not associated with depression symptoms (38). Elevated TPOAb levels cannot be used as a general marker of poor well-being or depression, as shown in a large population-based Danish study of 8,214 individuals (39).

With regard to bipolar disorder, sample size was small in several studies (40–43). Offspring of bipolar subjects were found more susceptible to develop thyroid autoantibodies independently from the susceptibility to develop psychiatric disorders (44). Autoimmune thyroiditis was suggested as a possible genetic biomarker for bipolar disorder in a twin study (45). Hashimoto’s encephalopathy can also occur with a bipolar disorder (46, 47), being vasculitis-related abnormalities in cortical perfusion one of the possible mechanisms (48). High levels of TPOAb were detected by Blanchin et al. (49) in the cerebrospinal fluid (CSF) of patients with Hashimoto’s encephalopathy. Both sera from their patients and monoclonal TPOAb were able to link monkey cerebellar cells. Moreover, normal human astrocytes from primary cultures binded monoclonal TPOAb. The presence of antigenic targets for anti-TSH-receptor IgG on human cortical neurons and TgAb IgG in cerebral vasculature was described by Moodley et al. (50).

## Postpartum Mood Disorders and Thyroid Autoimmunity

Risk for postpartum depression and alexithymia showed a direct borderline statistically significant correlation with serum TPOAb, suggesting that these mood disorders could be neurobehavioral consequences of an autoimmune attack (because of the TPOAb circulation in the CSF and of their possible cross-reaction with cerebral autoantigens) (3).

The PPT is more likely to occur in pregnant thyroid autoantibodies positive women compared to negative women (11). Most PPT women develop thyroid dysfunction during the first 6 months postpartum with initial mild symptoms of hyperthyroidism (heat intolerance, palpitations, weight loss, and fatigue) and a subsequent hypothyroid phase, frequently associated with depression (51). Approximately 50% of PPT women return euthyroid by 12 months PP (52).

Much uncertainty remains regarding the relationship between PPT and non-psychotic depression in thyroid autoantibodies positive euthyroid women. Harris et al. (53) found no difference in the rate of postpartum depression between thyroid autoantibodies positive women and thyroid autoantibodies negative women. However, the same author in 1992 reported an association between thyroid autoantibodies positivity and PPT (35). Subsequent studies on TPOAb positivity and postpartum depression in euthyroid women are controversial, founding no association (31, 54) or an association (34, 55). For instance, Kuijpens et al. studied prospectively 310 unselected women during gestation and up to 36 weeks postpartum (34). The presence of TPOAb was independently associated with depression at 12-week gestation and at 4 and 12 weeks postpartum (odds ratios between 2.4 and 3.8) in a prospective study on 310 unselected women (34).

A summary of the English-language literature in the last 25 years, which addressed the relationship between postpartum mood disorders and thyroid autoantibodies, is summarized in (Table 1).

**Table 1:** **Schematic comparison of the last 25 years studies on the relationship between postpartum mood disorders and thyroid autoantibodies**.

Reference	(56)	(57)	(58)	(35)	(34)	(59)	(3)
Country	USA	USA	Sweden	United Kingdom	The Netherlands	Greece	Italy
No. of women	51	119	27	242	291	55	74
Age (years)	18 or older	18–45	Not specified	26.6–25.9	30.8–29.5	32.6 ± 4.2	31.8 ± 4.64
Pregnancy evaluation	Yes	Yes	No	Yes	Yes	No	No
Postpartum evaluation	Month 1	Month 6	Day 5, week 6,	Week 6–8	Weeks 4, 12, 20, 28, 36	Day 1–4, week 4–6	Day 3
Maternity blues scale (and cutoff)	Not evaluated	Not evaluated	Month 6	Not evaluated	Not evaluated	PBQ (≥8.2)	Not evaluated
Depression scale (and cutoff score)	EPDS (≥13)	POMS (>20)	EPDS (≥12)	RDC EPDS (≥13) Hamilton (≥15) HAS HDS (≥11)	Not specified	EPDS (≥11)	EPDS (≥13; ≥14) MADRS (≥15)
Depression score, mean ± SD			6.3 ± 4.8, 5.8 ± 4.4, 5.1 ± 4.7				EPDS: 8.45 ± 4.4 MADRS: 14.3 ± 12.3
Depression rate	16.36%	POMS-D: 10.9%	15.3% (day 5) 11.7% (week 6) 11.5% (month 6)	RDC depression 47% TAb+ vs 32% TAb−, major depression 16% TAb+ vs 11% TAb− Hamilton 18% TAb+ vs 13% TAb− EPDS 39% TAb+ vs 27% TAb− HAS 34% TAb+ vs 30% TAb− HDS 22% TAb+ vs 17% TAb−	59% TPOAb+ vs 38% TPOAb−	24.19% (week 1) 22.8% (week 6)	EPDS (≥13): 20.3%, EPDS (≥14): 13.5% MADRS: 30%
Thyroid function	TSH, FT3, FT4	TSH	TSH, FT3, FT4	TSH, FT3, FT4	TSH, FT4	TSH, FT3, FT4	TSH, FT3, FT4
Thyroid antibodies	TgAb, TPOAb	TPOAb	TPOAb	Tab (not specified)	TPOAb	TgAb, TPOAb	TgAb, TPOAb
Correlation of PPD with thyroid indices	EPDS, TSH (*P* = 0.042) EPDS, TAb not specified (*P* = 0.043)	POMS, TPOAb+ (*P* = 0.023) POMS-D, TPOAb+ (*P* = 0.003) POMS-A, TPOAb+ (*P* = 0.013)	EPDS, FT3 (OR = 0.8) EPDS, TSH (OR = 11.30) EPDS, TPOAb (no association)	Hamilton, TAb+ (*P* = 0.0002) EPDS, Tab+ (*P* = 0.031) HDS, TAB+ (*P* = 0.003)	PPD%, TPOAb+ (*P* = 0.03)	Mood score, FT4 (rho −0.3, *P* ≤ 0.05) Mood score, TAb (no association)	EPDS, TPOAb^a^ (*P* = 0.056) EPDS, TgAb (*P* = 0.05)

*EPDS, Edinburgh Postnatal Depression Scale; GHQ, General Health Questionnaire; HAS, Hospital Anxiety Score; HDS, Hospital Depression Score; MADRS, Montgomery and Asberg Depression Rating Scale; OR, odds ratio; PBQ, Postpartum Blues Questionnaire; POMS, Profile of Mood States Scale (POMS-A, Anger; POMS-D, Depression); RDC, research diagnostic criteria; TgAb, thyroglobulin; TPOAb, thyroid autoantibodies against thyroperoxidase*.

*^a^Both TgAb and TPOAb levels were greater in depressed vs non-depressed women regardless of EPDS score threshold. Indeed, upon comparing score ≥13 vs score ≤12, TgAb was 38.5 ± 66.3 vs 16.6 ± 35.6 UI/ml (*P* = 0.05), and TPOAb was 26.2 ± 41.7 vs 10.5 ± 19.2 UI/ml (*P* = 0.05). Upon comparing score ≥14 vs score ≤13, TgAb was 29.9 ± 34.3 vs 19.7 ± 45.3 UI/ml (*P* = 0.045), and TPOAb was 34.3 ± 49.6 vs 10.5 ± 18.6 UI/ml (*P* = 0.023)*.

*The rate of TPOAb positiveness in the 10 women with an EPDS score ≥14 was 6.4-fold greater than in 64 women with an EPDS score ≥13 (30.0 vs 4.7%, χ^2^ = 7.437, *P* = 0.022)*.

In a follow-up study, Harris et al. (35) showed a significantly greater depression incidence in 110 thyroid autoantibodies positive women (47%) compared with 132 negative women (32%) regardless of thyroid dysfunction (Table 1). The same author in 2002 reported no difference in postpartum depression in TPOAb positive women treated with levothyroxine compared with TPOAb positive women given placebo and overall rates of major depression of 18.5% and depression in general of 38%, providing further evidence linking PPT and TPOAb positivity (60). No association was found between thyroid autoantibodies and postpartum mood disorders by Lambrinoudaki et al. (59). In another study, lower levels of serum FT3 were associated with increased incidence of mood disorders in the first postpartum week; only TPOAb and TgAb were significantly higher in women at risk for postpartum depression compared to women not at risk, using EPDS cutoff values of ≥13 or ≥14 (3). The presence of thyroid autoantibodies or higher TSH levels during the postpartum period may be related to depressive symptoms or dysphoric mood (56). Pregnant TPOAb positive women were shown to have higher depressive symptoms during pregnancy, and higher depression, anger, and total mood disturbance postpartum, regardless of the development of PPT (57).

Sylvén et al. (58) found an association between the TSH level over the clinical cutoff of 4.0 mU/l and the increased risk for depressive symptoms at 6 months postpartum in a Swedish population-based cohort (OR 11.30, 95% CI 1.93—66.11) (Table 1).

## Discussion

Tryptophan catabolites, indoleamine 2,3-dioxygenase, serotonin, and autoimmunity, as possible mediators of the immuno-inflammation consequences and the oxidative and nitrosative stress, may induce postpartum depression (61).

In summary, there is scanty literature on the relationship between postpartum mood disorders and thyroid autoantibodies, with data available for the United States and a few European countries. Furthermore, these available data stem from differing methodologies (e.g., psychometric scales, thyroid autoantibodies assays, time and frequency of measurements). Nevertheless, a direct, positive, unfavorable relationship does appear. Also studies evaluating the relationship between thyroid autoantibodies’ positivity and postpartum in euthyroid women have been mixed, with most of the studies demonstrating a significant association, confirmed also by a recent review of Dama et al. (62), who reported four of five studies finding a significant associations between TPOAb during gestation and postpartum depression (34–36, 57) and two of four studies finding links between postpartum TPOAb and depression (3, 34).

The cost-effectiveness of integrated perinatal mental health care has not been fully evaluated (63, 64) and is still controversial. The Edinburgh Postnatal Depression Scale (at a cut point of 16) had an incremental cost-effectiveness ratio (ICER) of £41,103 (€45,398, $67,130) per quality-adjusted life years (QALY) compared with routine care only. The ICER for all other strategies ranged from £49,928 to £272,463 per QALY vs routine care only, while the probability that no formal identification strategy was cost effective was 88% (59%) at a cost-effectiveness threshold of £20,000 (£30,000) per QALY. The major determinant of cost-effectiveness seems to be the potential additional costs of managing women incorrectly diagnosed as depressed (65). Leung et al. (66) showed that the use of EPDS as the screening tool and the provision of follow-up care had resulted in an improvement in maternal mental health at 6 months.

## Conclusion

Negative emotions during pregnancy should be recognized using self-report questionnaires as Profile of Mood States Scale, to select women at risk for deflected postpartum mood and requiring psychological support. However, more research is required to clarify the predictive value and pathophysiological implications of the associations between TgAb and/or TPOAb positivity and postpartum depression and to support solid evidence of benefit and cost-effectiveness of a careful neuropsychological evaluation in more 10% of all pregnant women (positive for TPO or Tg antibodies).

Future research on postpartum mood disorders should target genetic variations of the deiodinases, thyroid hormone transporters, and identification of central nervous system-expressed targets of TPOAb (or other coexisting Ab which are specifically directed against these targets), particularly in CNS areas engaged in depression and/or alexithymia.

## Author Contributions

All the authors have participated in drafting and revising the manuscript submitted whose contents they approve.

## Conflict of Interest Statement

The authors declare that the research was conducted in the absence of any commercial or financial relationships that could be construed as a potential conflict of interest.
